# Supplementary material for: NOTCH1 gene amplification promotes expansion of Cancer Associated Fibroblast populations in human skin
Source: Nat Commun. 2020 Oct 12;11:5126. doi: 10.1038/s41467-020-18919-2 (PMC7550609; doi:10.1038/s41467-020-18919-2)

Supplementary Information

NOTCH1 gene amplification promotes expansion of Cancer Associated Fibroblast populations in human skin.

Katarkar & Bottoni et al.

SUPPLEMENTARY FIGURE 1

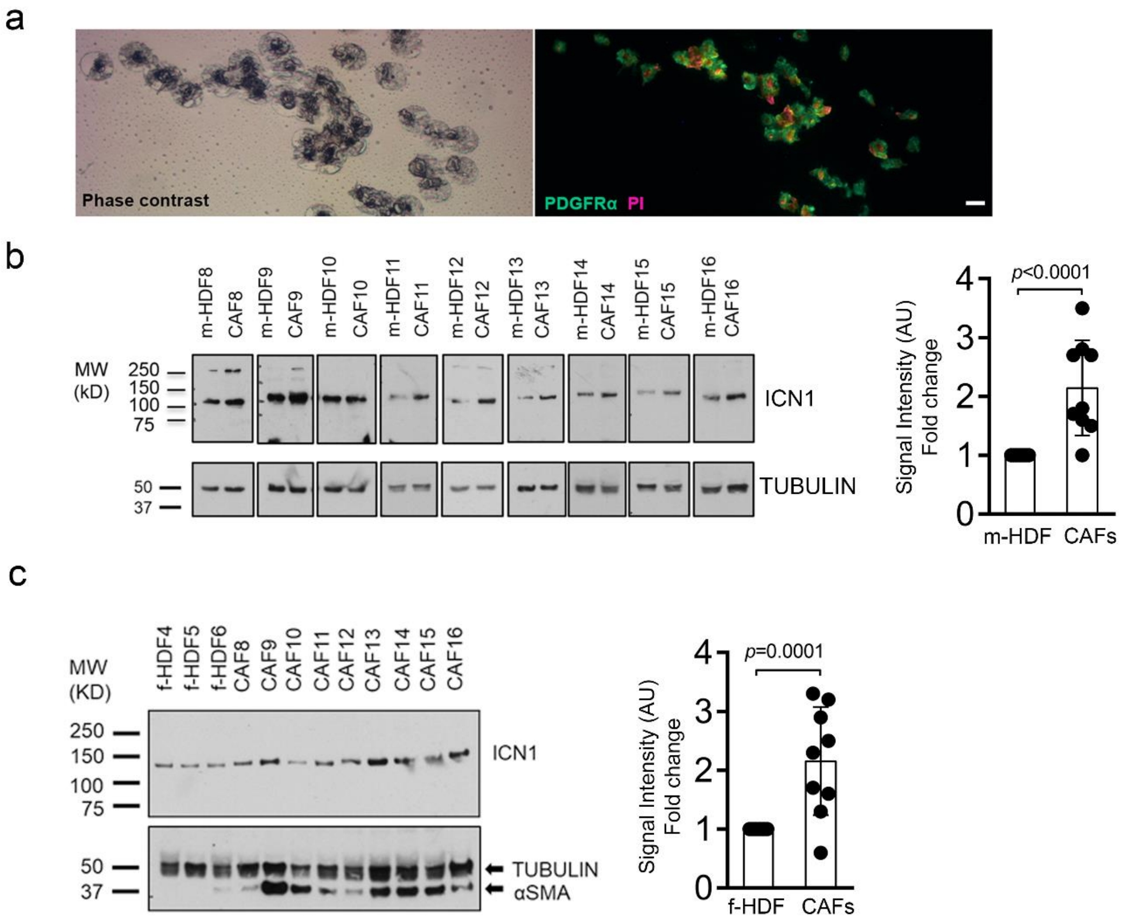

SUPPLEMENTARY FIGURE 1, related to FIGURE 2.
NOTCH1 overexpression in CAFs.
a, Representative images of phase contrast and PDGFRα positive fluorescence-guided laser capture microdissection (LCM) of fibroblasts (PDGFRα positive) from the same frozen blocks of SCC samples analyzed in Fig. 1d. Scale bar, 10 μm. **b**, Immunoblot analysis of different cultures of the same CAF strains as in Fig. 2f together with m-HDFs with antibodies against NOTCH1 and γ-TUBULIN together with densitometric quantification of ICN1 signal intensity after γ-TUBULIN normalization. Values for individual strains are indicated as dots with mean ± s.d, One sample t test, ****$p < 0.0001$, n(CAF strain) = 9, n(m-HDF strain) = 9, n(f-HDF strain) = 3. **c**, Immunoblot analysis of independent cultures of the same CAF strains as in Fig. 2f together with f-HDFs with antibodies against NOTCH1, α-SMA and γ-TUBULIN, together with densitometric quantification after γ-TUBULIN normalization. Values for each strain are indicated as dots with mean ± s.d, One sample t test, ***$p < 0.001$, n(CAF strain) = 9, n(f-HDF strain) = 3.

SUPPLEMENTARY FIGURE 2

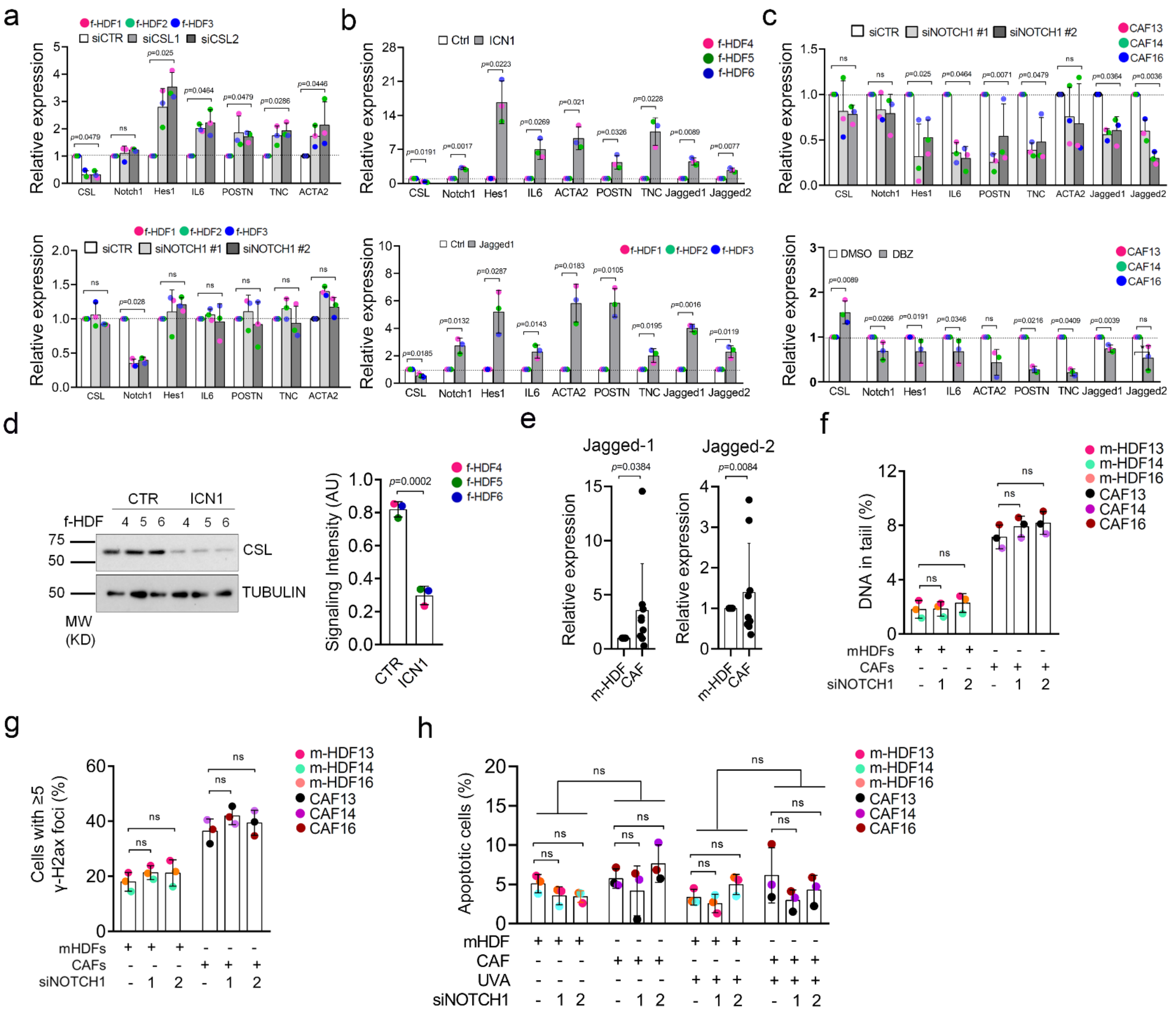

SUPPLEMENTARY FIGURE 2, related to FIGURE 3.

Consequences of modulation of NOTCH1 expression and activity in HDFs and CAFs.

a-c, Results of RT-qPCR analysis corresponding to the heat maps of Fig. 3a-c shown as individual plots. (**a**) f-HDF strains plus/minus CSL or NOTCH1 silencing by two different siRNAs versus control for 3 days. (**b**) f-HDF strains were infected with a lentivirus for doxycycline-inducible expression of ICN1 in parallel with empty vector control were treated with doxycycline for 5 days (upper panel) or cultured on dish pre-coated with immunoglobulin-coupled Jagged 1 ligand (1 µg/100ul) or immunoglobulins alone (Ctrl) for three days (lower panel). (**c**) CAF strains were infected with two lentiviruses for shRNA mediated NOTCH1 silencing in parallel with empty vector control (upper panel) or treated with the γ-Secretase Inhibitor DBZ (10 µM) versus DMSO vehicle alone for 5 days (lower panel). Values for each strain are indicated as dots with mean ± s.d. One way ANOVA (a, c upper panel) and One sample t test (b, c lower panel). *$p < 0.05$, **$p < 0.01$, ns = non-significant, n(f-HDF strain) = 3, n(CAF strain) = 3. **d**, Immunoblot analysis of CSL expression and γ-TUBULIN in f-HDF strains infected with an empty-vector versus ICN1-expressing lentiviruses. After selection f-HDFs were treated with doxycycline (500 ng/ml) for 5 days. n(f-HDF strain) = 3. Quantification of signal intensity of CSL normalized to γ-TUBULIN was shown as scatter plot, Values for each strain are indicated as dots with mean ± s.d, Two-tailed unpaired t-test, ***$p < 0.001$, n(f-HDF strain) = 3, **e**, RT-qPCR analysis of *JAGGED-1* and *JAGGED-2* mRNA expression in SCC-associated fibroblasts versus matched fibroblasts from flanking unaffected skin from the same samples as in Fig. 1d. Values for each strain are indicated as dots with mean ± s.d, One sample t test *$p < 0.05$, **$p < 0.01$, n(CAF strain) = 9, n(m-HDF strain) = 9. **f**, Comet assays in CAF and m-HDF strains plus/minus siRNA mediated NOTCH1 gene silencing. >148 cells were analyzed per condition. Values for each strain are indicated as dots with mean ± s.d, One way ANOVA with Dunnett's test, ns = non-significant, n(CAF strain) = 3, n(m-HDF strain) = 3. **g**, Immunofluorescence analysis of CAF and m-HDF strains plus/minus siRNA mediated NOTCH1 gene silencing (3 days) with antibodies against γ-H2AX staining. >200 cells were analyzed per condition. Values for each strain are indicated as dots with mean ± s.d, One way ANOVA with Dunnett's test, ns = non-significant, n(CAF strain) = 3, n(m-HDF strain) = 3. **h**, Quantification of apoptotic cells in CAF and m-HDF strains plus/minus siRNA mediated NOTCH1 gene silencing followed by UVA (500 mJ/cm2) treatment verses mock treated control (NT). >70 cells were analyzed per condition. Values for each strain are indicated as dots with mean ± s.d, Two-way ANOVA was performed between groups and two-tailed unpaired t-test was performed within each group, ns = non-significant, n(CAF strain) = 3, n(m-HDF strain) = 3.

SUPPLEMENTARY FIGURE 3

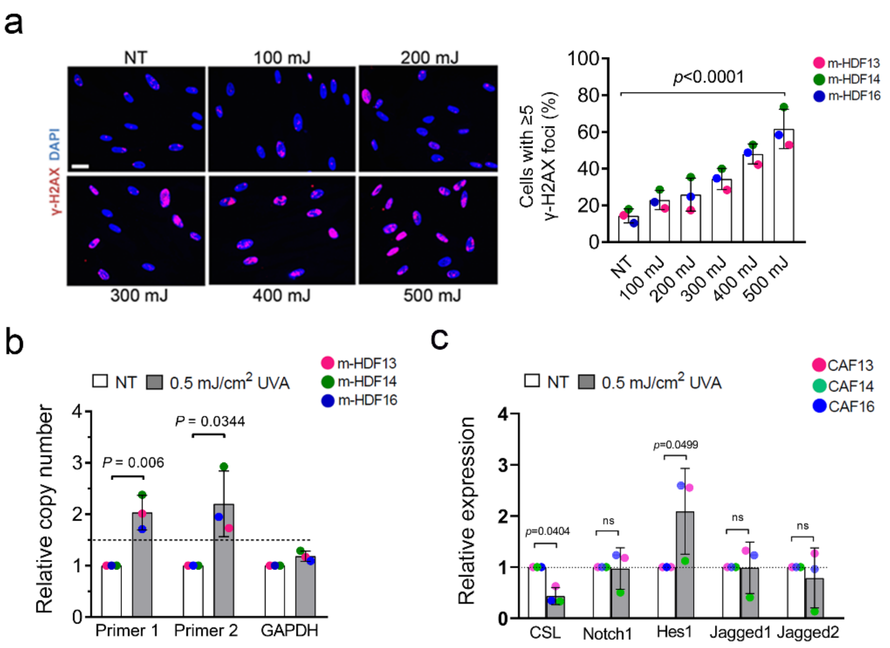

SUPPLEMENTARY FIGURE 3, related to FIGURE 4.
Expansion of dermal fibroblast populations with NOTCH1 gene amplification upon repeated UVA exposure.
a, Immunofluorescence analysis with γ-H2AX antibodies (red) in the same m-HDF strains plus/minus UVA treatment as in Fig. 4a. HDFs were irradiated with UVA (0, 100, 200, 300, 400, 500 mJ/cm2) every other day for 3 times and staining was performed 72 hours after the last exposure. Scale bar, 5 µm. >310 cells were analyzed per condition. Values for each strain are indicated as dots with mean ± s.d, One-way ANOVA, ***$p < 0.001$, *n*(m-HDF strain) = 3. **b**, Quantification of *NOTCH1* gene copy number in the same HDF strains from unaffected flanking skin used in Fig. 4a plus/minus UVA treatment. HDFs were irradiated with UVA (0, 500 mJ/cm2) every other day for 3 times and DNA was extracted 72 hours after the last exposure. Two primers for *NOTCH1* gene were used together with primers specific for the *GAPDH* gene used for internal normalization. DNA copies were calculated relative to the mock treated cells. Values for each strain are indicated as dots with mean ± s.d, One sample t test, *$p < 0.05$,**$p < 0.01$, *n*(m-HDF strain) = 3. **c**, RT-qPCR analysis of the indicated genes of f-HDF strains plus/minus UVA treatment as in Fig. 4c. Values for each strain are indicated as dots with mean ± s.d. One sample t test. *$p < 0.05$, ns = non-significant, *n*(f-HDF strain) = 3.

SUPPLEMENTARY FIGURE 4

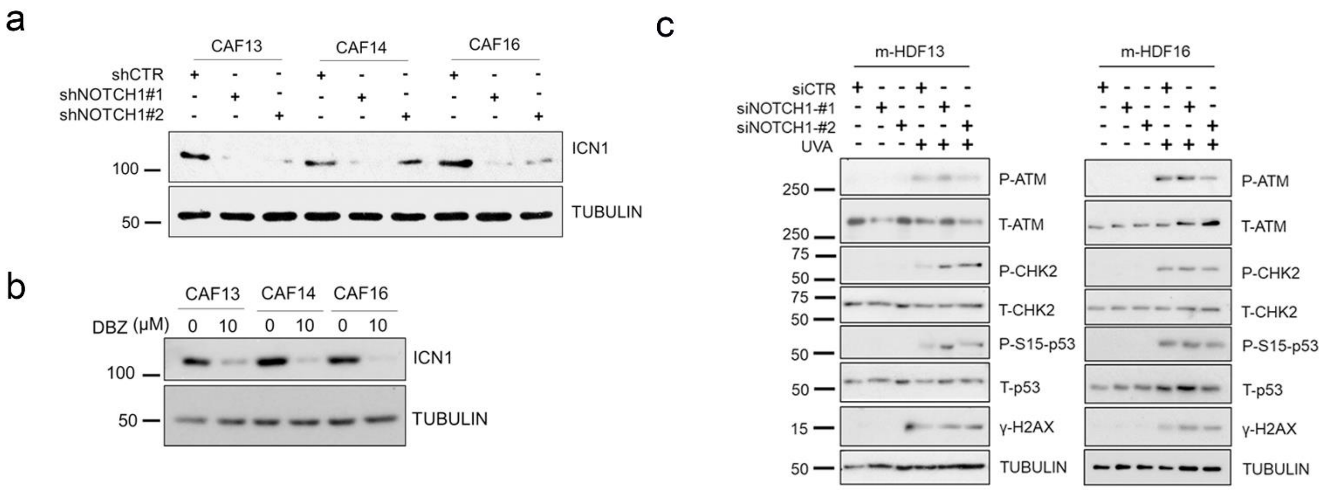

SUPPLEMENTARY FIGURE 4 related to FIGURE 6:
NOTCH1 expression after silencing with shRNA or inhibitors in CAFs
a, Immunoblot analysis of ICN1 expression and γ-TUBULIN in CAF strains infected with 2 *NOTCH1*-silencing lentiviruses versus control vector. *n*(CAF strain) = 3. **b**, Immunoblot analysis of ICN1 expression and γ-TUBULIN in CAF strains plus/minus DBZ treatment (10 µM). *n*(CAF strain) = 3. **c**, Immunoblot analysis of m-HDF strains plus/minus siNOTCH1 silencing and acute UVA treatment (500 mJ/cm2) versus mock treated with antibodies against P-ATM, P-CHK2, P-S15-p53 and corresponding total proteins, γ-H2AX and γ-TUBULIN. 24h after transfection with siRNAs, cells were treated with UVA and protein lysates were collected 24h after exposure. The same blots were stripped and re-probed. *n*(m-HDF) = 2.

SUPPLEMENTARY FIGURE 5

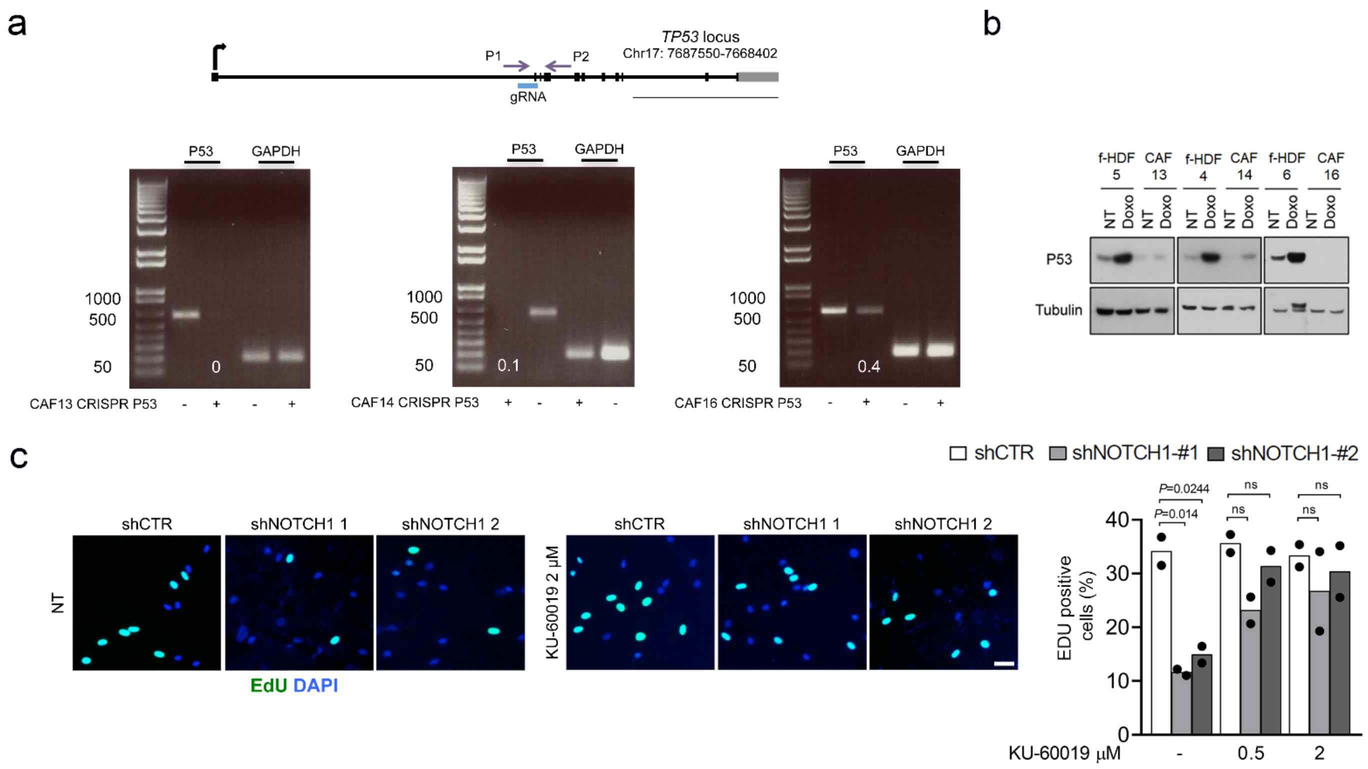

SUPPLEMENTARY FIGURE 5, related to FIGURE 7.

CRISPR-mediated TP53 deletion in CAFs; ATM inhibition impairs CAFs proliferation.

a, CRISPR-mediated TP53 deletion in CAFs. Top: Schematic representation of the TP53 locus with the position of the gRNA chosen for CRISPR/Cas9-mediated deletion and of the two primes (P1 and P2) used for PCR analysis. Bottom: The indicated CAF strains were infected with a CRISPR TP53 targeting vector as in [3], followed by selection for 7 days. PCR analysis was performed with a primer (P1) spanning the CRISPR-targeted TP53 disruption site (on exon 2). Numbers refer to residual detection of the TP53 gene in 3 CAF strains as assessed by densitometric quantification with GAPDH normalization. *n*(CAF strain) = 3. **b**, Immunoblot analysis with anti-p53 and γ-TUBULIN antibodies of CAFs with CRISPR-TP53 gene disruption in parallel with 3 f-HDF strain controls, plus/minus 24h treatment with Doxorubicin (1 µM) for stabilization of endogenous P53. *n*(CAF strain) = 3, *n*(f-HDF strain) = 3. **c**, EdU assays of CAF strains plus/minus NOTCH1 silencing for 7 days and additional 5 days of treatment with the ATM inhibitor (KU-60019, 0.5 or 2 µM) or DMSO vehicle alone. >152 cells were counted per sample. Values for each strain are indicated as dots with mean ± s.d, Two-tailed unpaired t-test, *$p < 0.05$, *n*(CAF strain) = 2.

SUPPLEMENTARY FIGURE 6

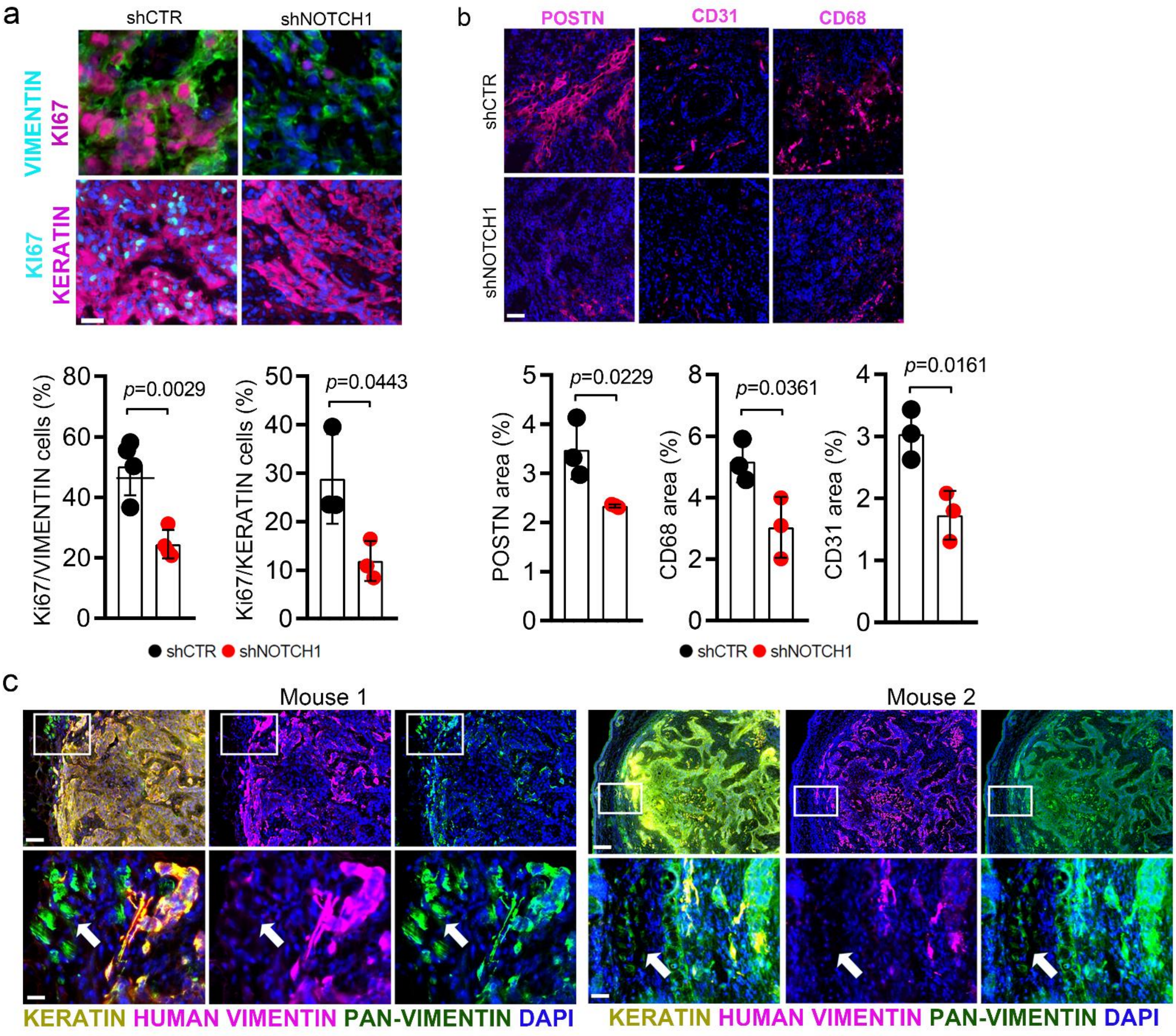

SUPPLEMENTARY FIGURE 6, related to FIGURE 9.

NOTCH1 silencing in CAFs impairs cancer/stromal cell expansion in vivo.

a, SCC13 cells were admixed with CAF16 plus/minus NOTCH1 silencing followed by parallel ear injections into NOD/SCID mice as in Fig. 9a. Shown are representative images and quantification of immunofluorescence analysis of multiple ear lesion pairs with antibodies against Ki67 and human-specific VIMENTIN (cyan) or KERATIN (magenta) for cell identification. Scale bar, 50 µm. Values for each ear lesions are indicated as dots with mean ± s.d, Two-tailed unpaired t-test, *p<0.05, **p<0.001, n(shCTR ear lesion) = 4, n(shNOTCH1 ear lesion) = 4 for VIMENTIN. n(mice) = 3, n(shCTR ear) = 3, n(shNOTCH1 ear) = 3 for KERATIN. **b**, Immunofluorescence analysis of ear lesions with the indicated antibodies. Scale bar, 50 µm. Values for each ear lesion are indicated as dots with mean ± s.d, Two-tailed unpaired t-test, *p<0.05, n(shCTR ear lesion) = 3, n(shNOTCH1 ear lesion) = 3. **c**, Representative low and high magnification images (scale bars, 100 and 10 µm) of ear injections as in a, stained with antibodies against KERATIN (yellow), PAN-Vimentin (green) and Human specific VIMENTIN (magenta).

SUPPLEMENTARY FIGURE 7

Fig. 2f

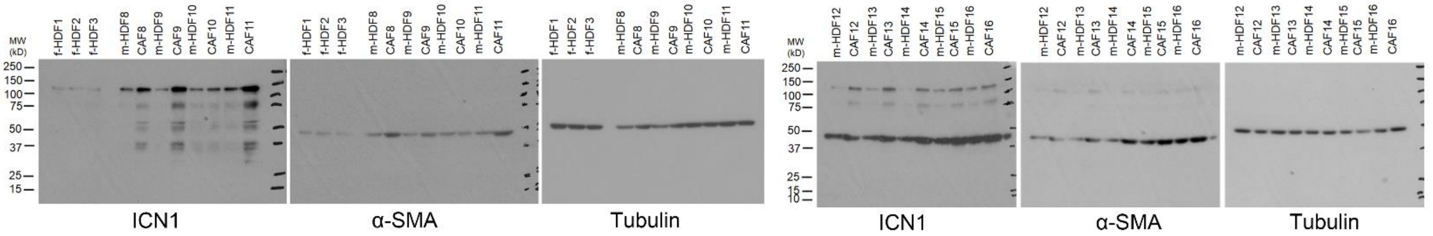

Extended data Fig. 1b

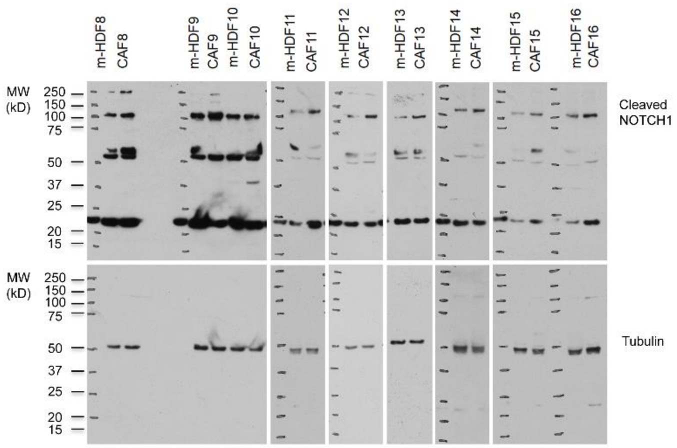

Extended data Fig. 1c

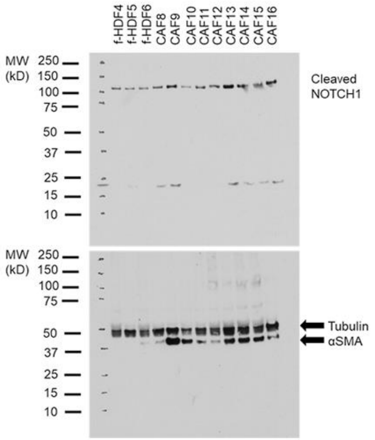

Extended data Fig. 2d

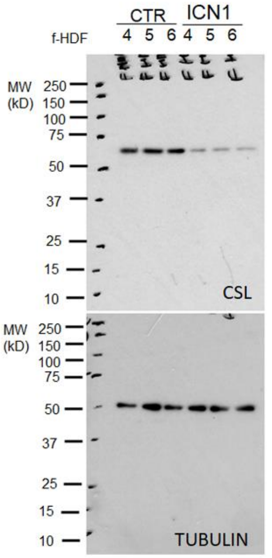

Fig. 5f

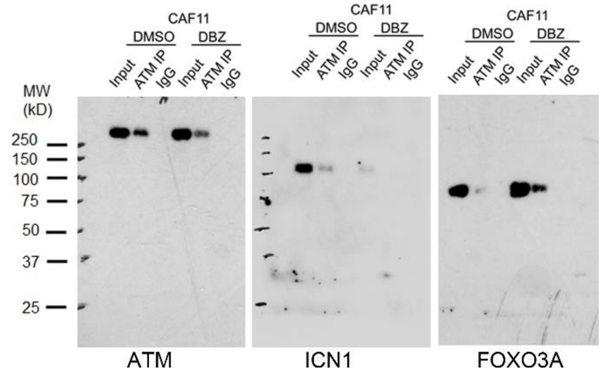

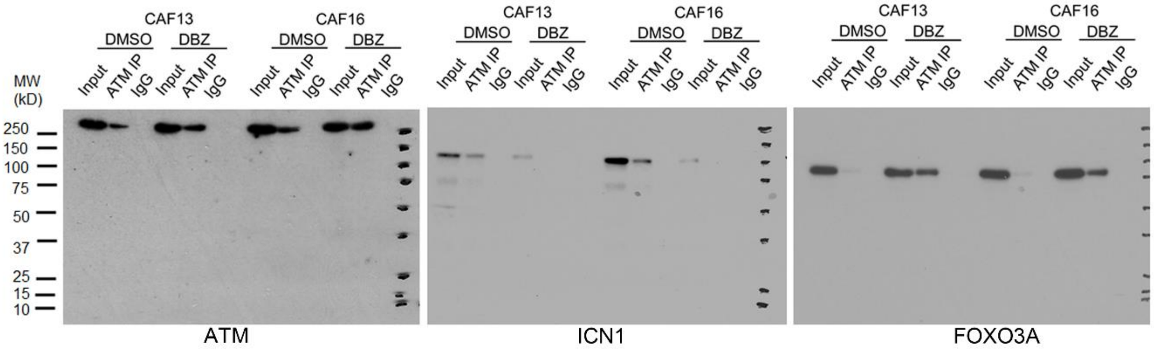

SUPPLEMENTARY FIGURE 8

Fig. 6c

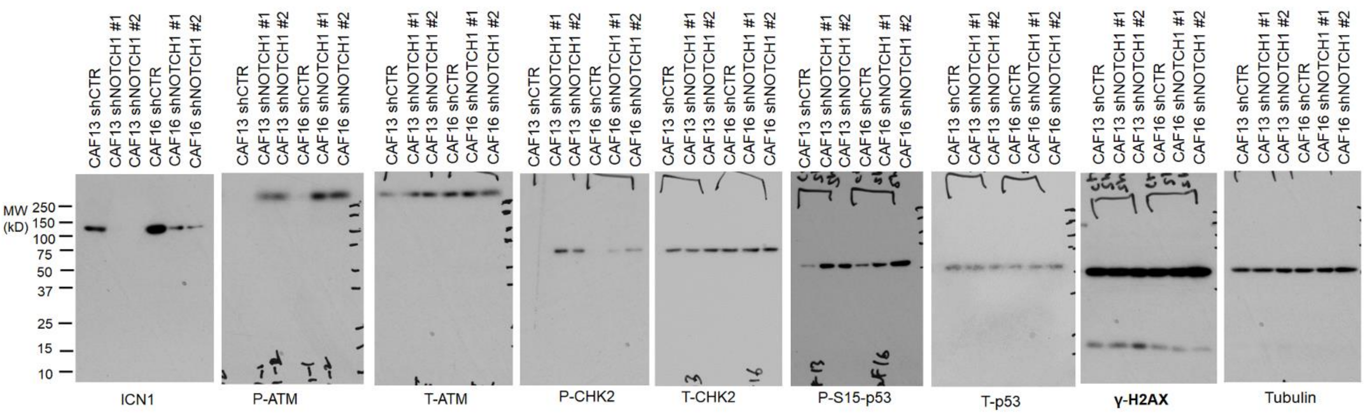

Fig. 6d

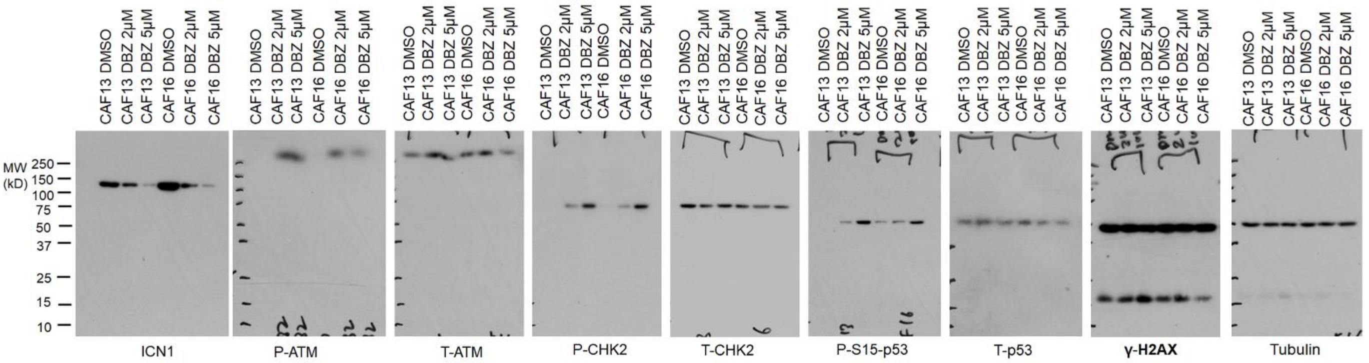

Fig. 6e

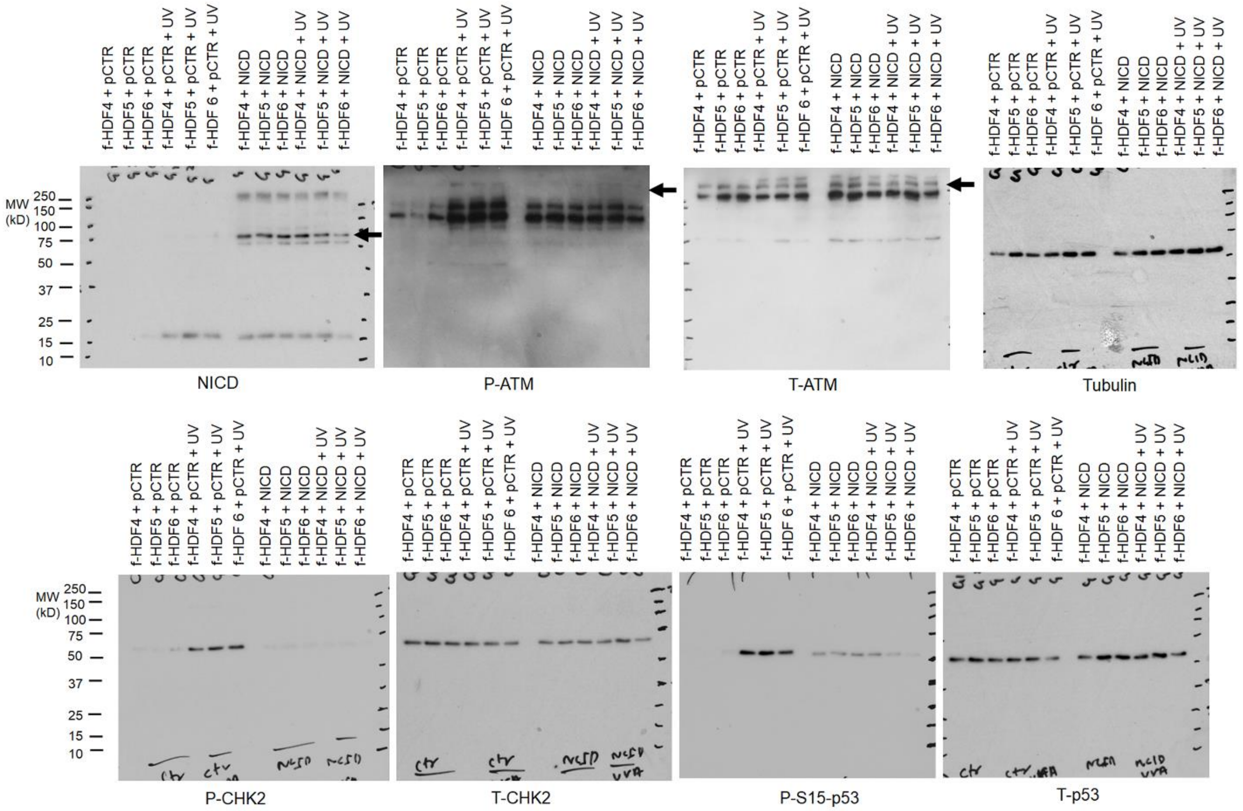

SUPPLEMENTARY FIGURE 9

Extended data Fig. 4a

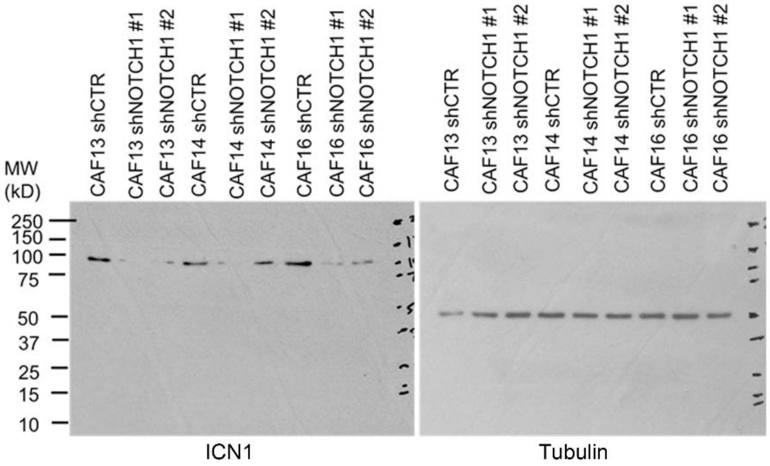

Extended data Fig. 4b

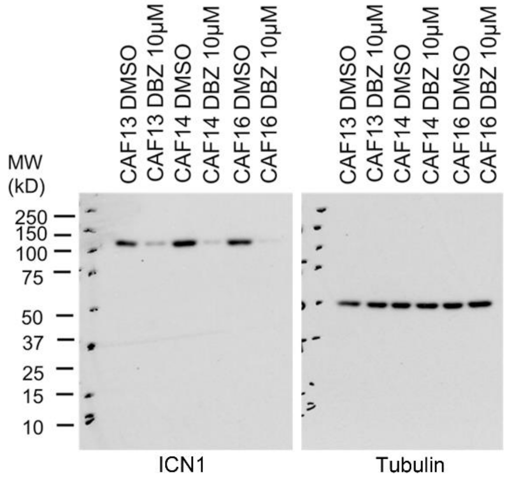

Extended data Fig. 4c

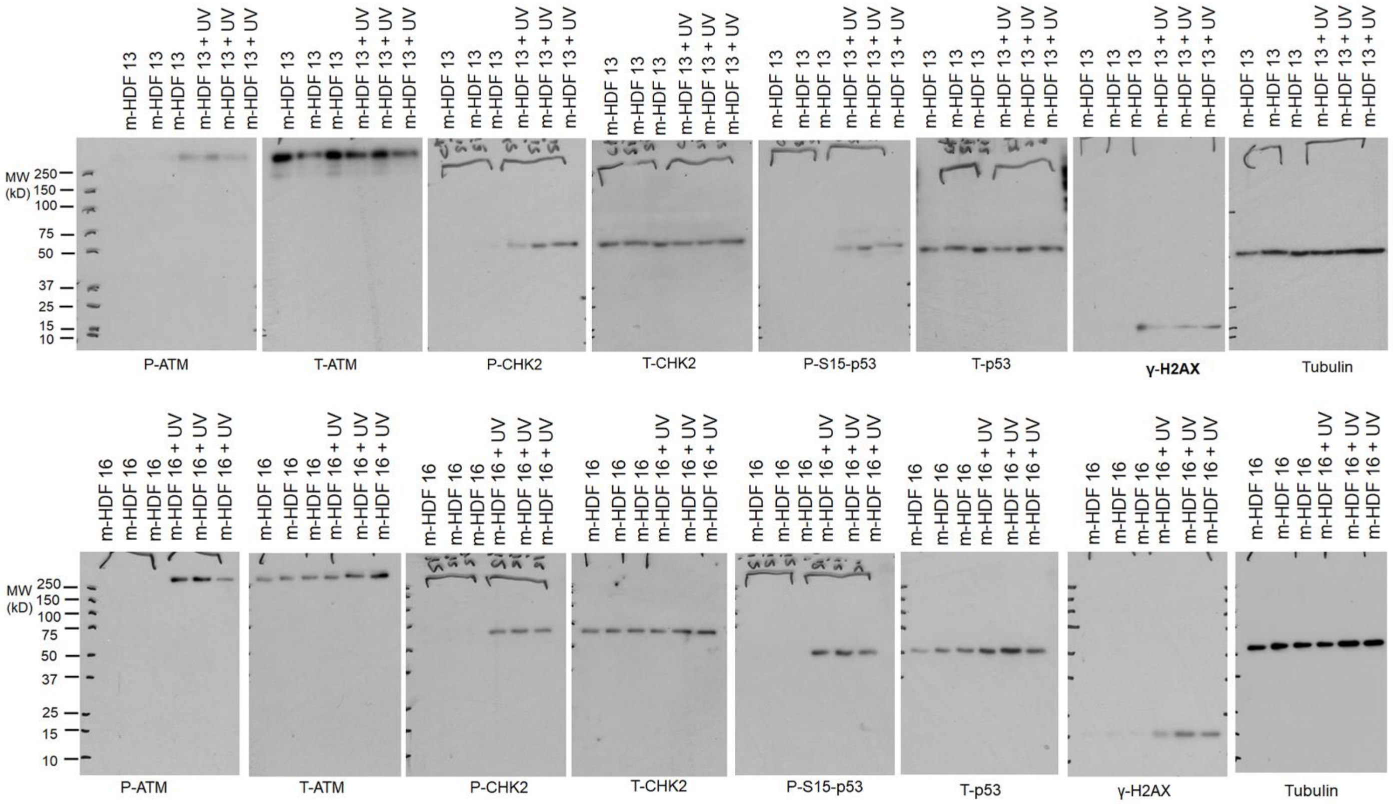

Extended data Fig. 5b

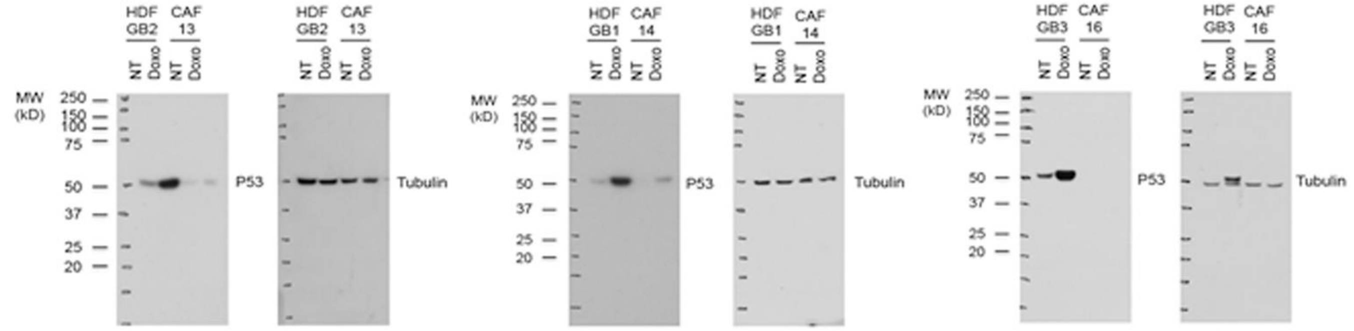

Supplement: Supplementary file 1 — Supplementary Information [file 41467_2020_18919_MOESM1_ESM.pdf]
