# Supplementary material for: NOTCH1 gene amplification promotes expansion of Cancer Associated Fibroblast populations in human skin
Source: Nat Commun. 2020 Oct 12;11:5126. doi: 10.1038/s41467-020-18919-2 (PMC7550609; doi:10.1038/s41467-020-18919-2)
Supplement: Supplementary file 3 — Description of Additional Supplementary Files [file 41467_2020_18919_MOESM3_ESM.pdf]

Description of Additional Supplementary Files

File Name: Supplementary Data 1
Description: List of regions amplified/deleted in CAFs following analysis of the aCGH data.

File Name: Supplementary Data 2-6
Description: List of cell strains, qPCR and RT-PCR oligonucleotides, silencer oligonucleotides and antibodies used in the study.
