# Supplementary material for: NOTCH1 gene amplification promotes expansion of Cancer Associated Fibroblast populations in human skin
Source: Nat Commun. 2020 Oct 12;11:5126. doi: 10.1038/s41467-020-18919-2 (PMC7550609; doi:10.1038/s41467-020-18919-2)
Supplement: Supplementary file 5 — Supplementary Data 2–6 [file 41467_2020_18919_MOESM5_ESM.pdf]

Supplementary Data 2. List of cell strains and identifiers.

**Data S2.<br>List of cell strains and identifiers**		
Cell type	Species	Name
Primary dermal fibroblasts	Human	f-HDF1
Primary dermal fibroblasts	Human	f-HDF2
Primary dermal fibroblasts	Human	f-HDF3
Primary dermal fibroblasts	Human	f-HDF4
Primary dermal fibroblasts	Human	f-HDF5
Primary dermal fibroblasts	Human	f-HDF6
Cancer associated fibroblasts	Human	CAF8
Cancer associated fibroblasts	Human	CAF9
Cancer associated fibroblasts	Human	CAF10
Cancer associated fibroblasts	Human	CAF11
Cancer associated fibroblasts	Human	CAF12
Cancer associated fibroblasts	Human	CAF13
Cancer associated fibroblasts	Human	CAF14
Cancer associated fibroblasts	Human	CAF15
Cancer associated fibroblasts	Human	CAF16
Matched dermal fibroblasts	Human	m-HDF8
Matched dermal fibroblasts	Human	m-HDF9
Matched dermal fibroblasts	Human	m-HDF10
Matched dermal fibroblasts	Human	m-HDF11
Matched dermal fibroblasts	Human	m-HDF12
Matched dermal fibroblasts	Human	m-HDF13
Matched dermal fibroblasts	Human	m-HDF14
Matched dermal fibroblasts	Human	m-HDF15
Matched dermal fibroblasts	Human	m-HDF16
SCC13	Human	RRID:CVCL_4029

Supplementary Data 3. Sequence of the oligonucleotides used for qPCR experiments.

**Data S3.<br>Sequence of the oligonucleotides used for qPCR experiments**		
Gene	Primer	Sequence
NOTCH1 set 1	F	AGCCTGCACAACCAGACAGA
	R	GCCACTGCCTACCTGGAAGA
NOTCH1 set 2	F	CAGGGTAACCAGGGCCTTC
	R	CCCTCGACAAAGCAACAGGT
NOTCH1 set 3	F	CCAACTGCGAGATCAACCTG
	R	TGGCTGACCTTGACCTCTGA
ERCC2	F	GATGCCAGCCCCTCTGAGT
	R	CACCTGAGCACCGTCTTCTG
UVSSA	F	CCTGCTTTAACACGCTTACCC
	R	CAGTGACGGAGGCAAGACTG
RPLP0	F	GAAATGTTTCATTGTGGGAGCA
	R	CCAGAGCTGGGTTGTTTTCC
GAPDH	F	CTGCCACCCAGAAGACTGTG
	R	GGTCAGGTCCACCACTGACA
TP53	F	CTTTCAGACTTCCTGAAAACAACGT
	R	GGGTGTGATGGGATGGATAAAAGC
TUBA1A	F	GAGTGCATCTCCATCCACGTT
	R	AGCCCCCGTCTCACTGAAG

Supplementary Data 4. List of antibodies used.

**Data S4.**<br>**List of antibodies used**				
Target	Species	Company and catalog #	Assay and dilution used	RRID
γ -H2AX	Rabbit	Cell Signaling #2577	IF (1:100), WB (1:1000)	RRID:AB_2118010
γ-Tubulin	Mouse	Sigma #GTU-88	WB (1:2000)	RRID:AB_477584
Vimentin	Rat	R&D # MAB2105 / Pan / Human/Mouse/Rat Vimentin	IF (1:200)	RRID:AB_2241653
Vimentin	Goat	Sigma # V4630-Human Specific	IF (1:20)	RRID:AB_477619
Cytokeratin	Mouse	BMA Biomedicals AG #T-1302	IF (1:200)	RRID:AB_1227343
P53	Mouse	Santa Cruz #126	WB (1:1000)	RRID:AB_628082
ATM	Mouse	Sigma #A1106	PLA (1:100), WB (1:1000)	RRID:AB_796190
FOXO3A	Rabbit	Cell Signaling #2497	PLA (1:100)	RRID:AB_836876
IgG	Mouse	Cell Signaling #5415	PLA (1:100)	RRID:AB_10829607
IgG	Rabbit	Cell signaling #2729	PLA (1:100)	RRID:AB_2617119
NOTCH1	Rabbit	Santa Cruz #6014	WB (1:1000), IF (1:100), PLA (1:100)	RRID:AB_650336
Anti-activated NOTCH1	Rabbit	Abcam #8925 (ICN1)	WB (1:1000), IF (1:100)	RRID:AB_306863
Cleaved Notch1 (Val1744) (D3B8)	Rabbit	Cell signaling #4147 (ICN1)	WB (1:1000), IF (1:100)	RRID:AB_2153348
PDGFRα	Goat	R&D #AF307NA	IF (1:100)	RRID:AB_354459
p-ATM	Rabbit	Cell signaling #13050	IF (1:50), WB (1:1000)	RRID:AB_2798100
p-CHK2	Rabbit	Cell signaling #2661	IF (1:50)	RRID:AB_331479
p-CHK2	Rabbit	Cell signaling #2197	WB (1:1000)	RRID:AB_2080501
pS/TQ	Rabbit	Cell signaling #2851	IF (1:50)	RRID:AB_330318
pS15-p53	Rabbit	Cell signaling #9284	IF (1:50), WB (1:1000)	RRID:AB_331464
Cytokeratin	Mouse	ENZO #ENZ-C34903	IF (1:200)	RRID:AB_2133885
POSTN	Rabbit	Abcam #92460	IF (1:100)	RRID:AB_2166645
CD68	Rat	Abcam #53444	IF (1:100)	RRID:AB_869007
CD31	Rat	BD/Pharmingen #550274	IF (1:100)	RRID:AB_393571
SMA	Mouse	Sigma #1A4	WB (1:2000)	RRID:AB_476701
CHK2	Rabbit	Cell signaling #6334	WB (1:1000)	RRID: AB_11178526
Ki67	Rabbit	GeneTex #GTX16667	IF (1:100)	RRID:AB_422351
CSL	Rabbit	Cell signaling #5313	WB (1:1000)	RRID:AB_2665555
ATM	Rabbit	Proteintech#27156-1-AP	IP (10 μg)	
Anti-Human IgG	Rabbit	Sigma # I2011		
Anti-rabbit Alexa fluor 568		Invitrogen # A10042	IF (1:1000)	
Anti-rat Alexa fluor 488		Invitrogen # A-11006	IF (1:1000)	
Anti-mouse Alexa fluor 568		Invitrogen # A32723	IF (1:1000)	
Anti-goat Alexa fluor 488		Invitrogen # A32814	IF (1:1000)	
VeriBlot for IP Detection HRP		Abcam #131366	WB (1:200)	

Supplementary Data 5. Sequence of the oligonucleotides used for qRT-PCR experiments.

**Data S5.<br>Sequence of the oligonucleotides used for qRT-PCR experiments**		
Gene	Primer	Sequence
RPLP0	F	GCAATGTTGCCAGTGTCTGT
	R	GCCTTGACCTTTTCAGCAAG
P53	F	AGGCCTTGGAACTCAAGGAT
	R	CTGAGTCAGGCCCTTCTGTC
P21	F	CCCAAGCTCTACCTTCCCAC
	R	ACAGGTCCACATGGTCTTCC
IL6	F	GTGTGAAAGCAGCAAAGAGGCACTG
	R	TGCCTTTTTCTGCAGGAACTGGATC
ACTA2	F	AGCGCAAATACTCTGTCTGG
	R	AGGCATAATTCCACAGGACA
POSTN	F	CAAAACTGAAGGACCCACAC
	R	TATTTCCACAGGCACTCCAT
TNC	F	TAACAGCATCACCCTGGAAT
	R	TCCTTGTCTTCCTTCACAGC
CSL	F	CAAAAGTTGCACAGAAGTCATA
	R	TGCTGCATTTCTTGGTCAC
NOTCH1	F	CTGAAGAACGGGGCTAACAA
	R	CAGGTTGTACTCGTCCAGCA
HES1	F	GGTGCTGATAACAGCGGAAT
	R	TGAGCAAGTGCTGAGGGTTT
JAGGED1	F	GGGAAAACGTGCCAGTTAGA
	R	ACAATTCTGACCCATCCAGC
JAGGED2	F	CAATGGTGGCATCTGTGTTG
	R	GCGATACCCGTTGATCTCAT

Supplementary Data 6. Identifiers for the Silencer™ oligonucleotides (Ambion and Thermofisher) used for RNA-interference experiments.

**Data S6.<br>Identifiers for the Silencer™ oligonucleotides (Ambion and Thermofisher) used for RNA-interference experiments**		
Gene name	#Catalog	#ID
Control	4390846	NA
NOTCH1 siRNA #1	4392420	9633
NOTCH1 siRNA #1	4392420	9634
CSL siRNA #1	4392420	7252
CSL siRNA #2	4392420	7253
