# Supplementary material for: NOTCH1 gene amplification promotes expansion of Cancer Associated Fibroblast populations in human skin
Source: Nat Commun. 2020 Oct 12;11:5126. doi: 10.1038/s41467-020-18919-2 (PMC7550609; doi:10.1038/s41467-020-18919-2)
Supplement: Supplementary file 6 — Reporting Summary [file 41467_2020_18919_MOESM6_ESM.pdf]

nature research

Corresponding author(s): Gian Paolo Dotto

Last updated by author(s): 26/07/2020

Reporting Summary

Nature Research wishes to improve the reproducibility of the work that we publish. This form provides structure for consistency and transparency in reporting. For further information on Nature Research policies, see Authors & Referees and the Editorial Policy Checklist .

Statistics

For all statistical analyses, confirm that the following items are present in the figure legend, table legend, main text, or Methods section.

n/a	Confirmed	
☐	☒	The exact sample size (*n*) for each experimental group/condition, given as a discrete number and unit of measurement
☐	☒	A statement on whether measurements were taken from distinct samples or whether the same sample was measured repeatedly
☐	☒	The statistical test(s) used AND whether they are one- or two-sided<br>*Only common tests should be described solely by name; describe more complex techniques in the Methods section.*
☐	☒	A description of all covariates tested
☐	☒	A description of any assumptions or corrections, such as tests of normality and adjustment for multiple comparisons
☐	☒	A full description of the statistical parameters including central tendency (e.g. means) or other basic estimates (e.g. regression coefficient) AND variation (e.g. standard deviation) or associated estimates of uncertainty (e.g. confidence intervals)
☐	☒	For null hypothesis testing, the test statistic (e.g. *F*, *t*, *r*) with confidence intervals, effect sizes, degrees of freedom and *P* value noted<br>*Give P values as exact values whenever suitable.*
☒	☐	For Bayesian analysis, information on the choice of priors and Markov chain Monte Carlo settings
☒	☐	For hierarchical and complex designs, identification of the appropriate level for tests and full reporting of outcomes
☒	☐	Estimates of effect sizes (e.g. Cohen's *d*, Pearson's *r*), indicating how they were calculated

Our web collection on statistics for biologists contains articles on many of the points above.

Software and code

Policy information about availability of computer code

Data collection	No softwere was used for data collection
Data analysis	Graph Pad PRISM 8, ImageJ 1.8, Feature Extraction software version 12 (Agilent Technologies), Agilent Genomic Workbench version 7 (Agilent Technologies)

For manuscripts utilizing custom algorithms or software that are central to the research but not yet described in published literature, software must be made available to editors/reviewers. We strongly encourage code deposition in a community repository (e.g. GitHub). See the Nature Research guidelines for submitting code & software for further information.

Data

Policy information about availability of data

All manuscripts must include a data availability statement. This statement should provide the following information, where applicable:

- Accession codes, unique identifiers, or web links for publicly available datasets
- A list of figures that have associated raw data
- A description of any restrictions on data availability

GSE113577 (Fig. 1a), Source data for Figures 1b-d, 2a-f, 3a-h, 4a-d, 5a-f, 6a-e, 7a-f, 8a-b, 9a-d, 10a-d and Supplementary Figures 1b-c, 2a-h, 3a-c, 4a-c, 5b-c, 6a-b are provided as a Source Data file.

Field-specific reporting

Please select the one below that is the best fit for your research. If you are not sure, read the appropriate sections before making your selection.

Life sciences study design

All studies must disclose on these points even when the disclosure is negative.

Sample size	No statistical method was used to predetermine sample size. The samples sizes were selected based on previous studies with similar methodologies. Bottoni, G.,Nat Commun 10, 3884 (2019). https://doi.org/10.1038/s41467-019-11785-7
Data exclusions	No inclusion / exclusion criteria were adopted.
Replication	All attempts at replication were successful and mentioned in the figure legends.
Randomization	No randomization was adopted since matched pairs of the HDFs, CAFs +/- specific genetic modifications were used for the study
Blinding	No blinding was adopted since matched pairs of the HDFs, CAFs +/- specific genetic modifications were used for the study

Reporting for specific materials, systems and methods

We require information from authors about some types of materials, experimental systems and methods used in many studies. Here, indicate whether each material, system or method listed is relevant to your study. If you are not sure if a list item applies to your research, read the appropriate section before selecting a response.

Materials & experimental systems

n/a	Involved in the study	
☐	☒	Antibodies
☐	☒	Eukaryotic cell lines
☒	☐	Palaeontology
☐	☒	Animals and other organisms
☐	☒	Human research participants
☒	☐	Clinical data

Methods

n/a	Involved in the study	
☒	☐	ChIP-seq
☒	☐	Flow cytometry
☒	☐	MRI-based neuroimaging

Antibodies

Antibodies used

γ -H2AX Rabbit Cell Signaling #2577 IF (1:100), WB (1:1000) RRID:AB_2118010
γ-Tubulin Mouse Sigma #GTU-88 WB (1:2000) RRID:AB_477584
Vimentin Rat R&D # MAB2105 / Pan / Human/Mouse/Rat Vimentin IF (1:200) RRID:AB_2241653
Vimentin Goat Sigma # V4630-Human Specific IF (1:20) RRID:AB_477619
Cytokeratin Mouse BMA Biomedicals AG #T-1302 IF (1:200) RRID:AB_1227343
P53 Mouse Santa Cruz #126 WB (1:1000) RRID:AB_628082
ATM Mouse Sigma #A1106 PLA (1:100), WB (1:1000) RRID:AB_796190
FOXO3A Rabbit Cell Signaling #2497 PLA (1:100) RRID:AB_836876
IgG Mouse Cell Signaling #5415 PLA (1:100) RRID:AB_10829607
IgG Rabbit Cell signaling #2729 PLA (1:100) RRID:AB_2617119
NOTCH1 Rabbit Santa Cruz #6014 WB (1:1000), IF (1:100), PLA (1:100) RRID:AB_650336
Anti-activated NOTCH1 Rabbit Abcam #8925 (ICN1) WB (1:1000), IF (1:100) RRID:AB_306863
Cleaved Notch1 (Val1744) (D3B8) Rabbit Cell signaling #4147 (ICN1) WB (1:1000), IF (1:100) RRID:AB_2153348
PDGFRα Goat R&D #AF307NA IF (1:100) RRID:AB_354459
P-ATM Rabbit Cell signaling #13050 IF (1:50), WB (1:1000) RRID:AB_2798100
P-CHK2 Rabbit Cell signaling #2661 IF (1:50) RRID:AB_331479
P-CHK2 Rabbit Cell signaling #2197 WB (1:1000) RRID:AB_2080501
pS/TQ Rabbit Cell signaling #2851 IF (1:50) RRID:AB_330318
pS15-p53 Rabbit Cell signaling #2851 IF (1:50), WB (1:1000) RRID:AB_331464
Cytokeratin Mouse ENZO #ENZ-C34903 IF (1:200) RRID:AB_2133885
POSTN Rabbit Abcam #92460 IF (1:100) RRID:AB_2166645
CD68 Rat Abcam #53444 IF (1:100) RRID:AB_869007
CD31 Rat BD/Pharmingen #550274 IF (1:100) RRID:AB_393571
SMA Mouse Sigma #1A4 WB (1:2000) RRID:AB_476701
CHK2 Rabbit Cell signaling #6334 WB (1:1000) RRID:AB_869138
Anti-Human IgG Rabbit Sigma # I2011
Ki67 Rabbit GeneTex #GTX16667 IF (1:100) RRID:AB_422351
CSL Rabbit Cell signaling #5313 WB (1:1000) RRID:AB_2665555
ATM Rabbit Proteintech#27156-1-AP IP (10 μg)

Anti-rabbit Alexa fluor 568 Invitrogen # A10042 IF (1:1000)
Anti-rat Alexa fluor 488 Invitrogen # A-11006 IF (1:1000)
Anti-mouse Alexa fluor 568 Invitrogen # A32723 IF (1:1000)
Anti-goat Alexa fluor 488 Invitrogen # A32814 IF (1:1000)
VeriBlot for IP Detection HRP Abcam #131366 WB (1:200)

Validation

Information of Anti-phospho-γ -H2AX Rabbit Cell Signaling #2577 for IF and WB can be found at product website: https://www.cellsignal.com/products/primary-antibodies/phospho-histone-h2a-x-ser139-antibody/2577

Information of Anti-γ-Tubulin Mouse Sigma #GTU-88 for WB can be found at product website; https://www.sigmaaldrich.com/catalog/product/sigma/t6557?
lang=fr&region=CH&gclid=Cj0KCQjw7ZL6BRCmARIsAH6XFDL98YHf2xBhgRgaT4m0pyX6fCr7ziELkUzMj-miHfb7DejmkVYBNzQaAnGoEALw_wcB

Information of Anti-Vimentin Rat R&D # MAB2105 /Pan /Human/Mouse/Rat Vimentin for IF can be found at product website; '(https://www.rndsystems.com/products/human-mouse-rat-vimentin-antibody-280618_mab2105#product-details

Information of Anti-Vimentin Goat from Sigma # V4630-Human Specific for IF can be found at product website; https://www.sigmaaldrich.com/catalog/product/sigma/v4630?lang=fr&region=CH

Information of Anti Cytokeratin, Mouse from BMA Biomedicals AG #T-1302 for IF can be found at product website; https://www.dianova.com/en/shop/t-1302-anti-cytokeratin-pan-huve-from-mouse-lu-5-unconj/

Information of Anti -P53 from Santa Cruz #126 WB (1:1000) for WB can be found at product website; https://www.scbt.com/p/p53-antibody-do-1

Information of Anti-ATM antibody, Mouse monoclonal for WB can be found at product website; https://www.sigmaaldrich.com/catalog/product/sigma/a1106?lang=fr&region=CH and we also validate for proximity ligation assay.

Information of Anti-FOXO3A Rabbit from Cell Signaling #2497 for WB can be found at product website; https://www.cellsignal.com/products/primary-antibodies/foxo3a-75d8-rabbit-mab/2497

Information of anti-Cleaved Notch1 (Val1744) (D3B8) Rabbit mAb #4147 for WB can be found at product website; https://www.cellsignal.com/products/primary-antibodies/cleaved-notch1-val1744-d3b8-rabbit-mab/4147, also we have validate the antibody in CAFs +/- shNOTCH1 silencing as well as for the IF detection.

Information of Anti-activated NOTCH1, Rabbit Abcam #8925 (ICN1) for WB can be found at product website; https://www.abcam.com/activated-notch1-antibody-ab8925.html.

Information of anti-NOTCH1 Rabbit from Santa Cruz #6014 for several application can be found here, https://www.citeab.com/antibodies/819223-sc-6014-notch-1-antibody-c-20

Information of anti-PDGFRα R&D #AF307NA can be found at product website; https://www.rndsystems.com/products/human-pdgf-ralpha-antibody_af-307-na

Information of Anti- p-ATM Rabbit from Cell signaling #13050 for WB can be found at product website; https://www.cellsignal.com/products/primary-antibodies/phospho-atm-ser1981-d25e5-rabbit-mab/13050?site-search-type=Products, we also validate the antibody for IF analysis.

Information Anti-p-CHK2 Rabbit from Cell signaling #2661 for IF can be found at product website; https://www.cellsignal.com/products/primary-antibodies/phospho-chk2-thr68-antibody/2661, we also validate the antibody for IF analysis.

Information of Anti-p-CHK2 Rabbit from Cell signaling #2197 for IWB can be found at product website; https://www.cellsignal.com/products/primary-antibodies/phospho-chk2-thr68-c13c1-rabbit-mab/2197.

Information of Anti-pS/TQ Rabbit from Cell signaling #2851 for WB can be found at product website; https://www.cellsignal.com/products/primary-antibodies/phospho-ser-thr-atm-atr-substrate-antibody/2851, we also validate the antibody for IF analysis.

Information of Anti- pS15-p53 Rabbit from Cell signaling #9284 for WB can be found at product website; https://www.cellsignal.com/products/primary-antibodies/phospho-p53-ser15-antibody/9284, we also validate the antibody for IF analysis.

Information of Anti- Cytokeratin Mouse from ENZO #ENZ-C34903 for IF can be found at product website; https://www.cellsignal.com/products/primary-antibodies/phospho-p53-ser15-antibody/9284.

Information of Anti- POSTN Rabbit from Abcam #92460 for IF can be found at product website; https://www.abcam.com/periostin-antibody-ab14041.html.

Information of Anti- CD68 Rat from Abcam #53444 for IF can be found at product website; https://www.abcam.com/periostin-antibody-ab14041.html.

Information of Anti- CD31 Rat from BD/Pharmingen #550274 for IF can be found at data sheet; https://www.bdbiosciences.com/ds/pm/tds/550274.pdf.

Information of Anti- SMA Mouse from Sigma #1A4 for WB can be found at website; https://www.sigmaaldrich.com/catalog/product/sigma/a5228?lang=fr&region=CH.

Information of Anti- SMA Mouse from Sigma #1A4 for WB can be found at website; https://www.cellsignal.com/products/primary-antibodies/chk2-d9c6-xp-rabbit-mab/6334.

Information of Anti- CHK2 Rabbit from Cell signaling #6334 for WB can be found at website; https://www.sigmaaldrich.com/catalog/product/sigma/a5228?lang=fr&region=CH.

Information of Anti- Ki67 Rabbit from GeneTex #GTX16667 for IF can be found at website; https://www.genetex.com/Product/Detail/Ki67-antibody-SP6/GTX16667.

Information of Anti- CSL Rabbit from Cell signaling #5313 for WB can be found at website; https://www.cellsignal.com/products/primary-antibodies/rbpsuh-d10a4-xp-rabbit-mab/5313.

Information of Anti- ATM Rabbit from Proteintech#27156-1-AP for WB and IP analysis can be found at website; https://www.ptglab.com/products/ATM-Antibody-27156-1-AP.htm.

Information of VeriBlot for IP Detection Reagent (HRP) (ab131366) for WB can be found at website; https://www.abcam.com/veriblot-for-ip-detection-reagent-hrp-ab131366.html.

All the antibodies used in the study were previously tested and published by the authors or other groups.

Eukaryotic cell lines

Policy information about cell lines

Cell line source(s)	HDFs were prepared from discarded foreskin or abdominoplasty skin samples at the Department of Dermatology, Massachusetts General Hospital (Boston, Massachusetts, USA) with institutional approval (2000P002418), or were previously obtained. Pairs of CAFs and matched HDFs from discarded skin SCC and flanking unaffected areas from the same (anonymized) patients were given specific identifiers.
Authentication	HDF and CAF strains were tested in IF to be Vimentin-positive and Keratin-negative.
Mycoplasma contamination	All cell lines tested negative for mycoplasma contamination
Commonly misidentified lines (See ICLAC register)	No commonly misidentified cell lines were used in the study.

Animals and other organisms

Policy information about studies involving animals; ARRIVE guidelines recommended for reporting animal research

Laboratory animals	Mouse ear injections of cells were carried out in 8 to 10-week-old female NOD/SCID/IL2rγ−/− mice. The mouse was kept in the standard housing condition following 12 light/12 dark cycle and temperatures of 65-75°F (~18-23°C) with 40-60% humidity.
Wild animals	No wild animals were used in the study.
Field-collected samples	No field collected samples were used in the study.
Ethics oversight	All animal studies were approved by the Massachusetts General Hospital Institutional Animal Care and Use Committee (2004N000170) or were performed according to the Swiss guidelines and regulations for the care and use of laboratory animals, with approved protocol from the Canton de Vaud veterinary office (animal license No. 1854.4e).

Note that full information on the approval of the study protocol must also be provided in the manuscript.

Human research participants

Policy information about studies involving human research participants

Population characteristics	*Describe the covariate-relevant population characteristics of the human research participants (e.g. age, gender, genotypic information, past and current diagnosis and treatment categories). If you filled out the behavioural & social sciences study design questions and have nothing to add here, write "See above."*
Recruitment	*Describe how participants were recruited. Outline any potential self-selection bias or other biases that may be present and how these are likely to impact results.*
Ethics oversight	*Identify the organization(s) that approved the study protocol.*

Note that full information on the approval of the study protocol must also be provided in the manuscript.
